# A Review, Update, and Commentary for the Cough without a Cause: Facts and Factoids of the Habit Cough

**DOI:** 10.3390/jcm12051970

**Published:** 2023-03-02

**Authors:** Miles Weinberger, Dennis Buettner, Ran D. Anbar

**Affiliations:** 1 Rady Children’s Hospital, University of California San Diego, San Diego, CA 92123, USA; 2 University of Iowa, Iowa City, IA 50011, USA; 3 Habit Cough Association, Severna Park, MD 21146, USA; 4 Center Point Medicine, La Jolla, CA 92037, USA

**Keywords:** cough, chronic cough, habit cough, suggestion therapy, hypnosis, behavioral therapy, clinical education

## Abstract

Background: A habitual cough, persisting after the cause is gone, was described in a 1694 medical book. Successful treatment of this disorder known as habit cough was reported in 1966 by the “art of suggestion”. The purpose of this article is to provide the current basis for diagnosis and treatment of the Habit Cough Syndrome. Method: The epidemiology and clinical course of habit cough were reviewed; original data were obtained from three sources. Results: Unique clinical presentation was the basis for diagnosis of habit cough. Diagnosis was made 140 times with increasing frequency over 20 years at the University of Iowa clinic and 55 times over 6 years at a London clinic. Suggestion therapy provided more frequent cessation of cough than just reassurance. A Mayo Clinic archive of chronic involuntary cough found 16 of 60 still coughing 5.9 years after initial evaluation. Ninety-one parents of children with habit cough and 20 adults reported cessation of coughing from viewing a publicly available video of successful suggestion therapy. Conclusions: Habit cough is recognizable from the clinical presentation. It is effectively treated in most children by suggestion therapy in clinics, by remote video conferencing, and by proxy from viewing a video of effective suggestion therapy.

## 1. Introduction

“*The morbidity, cost, and impact of somatoform (functional) pediatric respiratory syndromes are far more impactful than that experienced by most of our severe asthmatics and CF patients. These conditions are NOT trivial, ‘just in your head’ conditions. A confident diagnostic approach and an optimistic therapeutic regimen are critically important.*” (George Mallory MD)

Physician education focuses on anatomical, physiological, and organic disorders that cause illness. Emphasis is on identifying what is wrong with the body that is causing the patient’s symptoms. When the cause is not identified, medication typically is provided in an attempt to relieve the symptoms. But some symptoms are not caused by corporal dysfunction and do not respond to usual pharmacotherapy. The medical term for those is functional. Among the clinically important functional respiratory disorders is the habit cough [1]. Habit cough has a long history in the medical literature. Unfortunately, habit cough currently often has been associated with inadequate recognition and alternative diagnoses. 

The purpose of this article is to promote increased awareness among physicians regarding this often misdiagnosed and overtreated disorder. Habit cough is not influenced by any pharmacotherapy but does respond, with rare exceptions, to a simple behavioral measure. This narrative of the history, current status, and barriers to physician education regarding habit cough includes the encounters and accumulated data from the three authors who have an extraordinary amount of exposure to this disorder. We will review the chronology, epidemiology, etiology, treatment, and educational efforts to increase the understanding of this disorder. We will also describe the efforts to increase the knowledge and clinical skills of physicians likely to encounter this disorder. However, we cannot discuss habit cough without also examining the prevailing controversies and misinformation regarding this functional disorder, the cough without a cause.

## 2. Methods

Three present sources of data were used to characterize habit cough: (1) The University of Iowa Pediatric Allergy and Pulmonary Clinic from 1994 to 2014 provided epidemiologic data not previously published. (2) Over 200 remote contacts from February 2019 to September 2022 provided insights and aspects of outcome beyond what could be derived from clinic patients. (3) Selected patients from a private practice of author RA provided observational and treatment data about children with recalcitrant habit cough. Data from these sources will be contrasted with guidelines for cough management from authoritative committees.

The three sources of habit cough used for this publication provide data on the successes and limitations of suggestion therapy for habit cough as direct treatment, indirect treatment by remote video conferencing, and treatment by proxy where a video of a child receiving suggestion therapy is the source of successful suggestion therapy in others. From these data sources, epidemiology, outcome, and phenotypical variations of habit cough can be extracted.

Recognizing the relative deficiency of knowledge about habit cough and its treatment outside of those of us with extensive exposure, present physician care of habit cough also was available from almost 4 years of remote contact. Phenotypical variations of intractable habit cough and their treatment were available from the hypnosis and counseling practice of author RA.

## 3. Results

### 3.1. Chronology of Habit Cough

We found medical books from the 17th and 19th centuries with descriptions of specific examples of chronic cough that are consistent with current observations of habit cough. A medical textbook from 1685 by Thomas Willis (1621–1675) [2] described an adult woman with “*a violent dry cough following her day and night, unless she was fallen asleep*”, a description that would fit the habit cough diagnosis today. Franciscus Mercurios (1614–1699), a Flemish physician, alchemist, kabbalist, and writer, in a 1694 medical book, *The Spirit of Diseases*, described, “*…Habitual Cough, which often continues after the first cough, which was caused by the cold, is gone… and the Habitual Cough often proceed*” [3]. Charles Creighton (1847–1927), a British physician and medical author, in an 1886 medical book, *Illustrations of Unconscious Memory in Disease*, described, “*…a habit cough—a reflex effect persisting after the cause is gone… or an acquired habit…*” He went on to state, “*…the treatment of it is to break the habit…*” [4]. This was insightful reasoning on the part of Dr. Creighton.

Breaking the habit was essentially what Dr. Bernard Berman, a Boston allergist, reported in a 1966 publication [5]. He described six children seen in his practice over a 5-year period, three boys and three girls between the ages of 9 and 13 years. They all had daily barking coughing for the prior 3–6 months with absence of cough when asleep. He concluded that they were “*afflicted with habit cough*”; Dr. Berman stopped the coughing by what he called “*a simple modality of therapy*” that required an “*understanding and experience in the art of suggestion*”. Being aware that this repetitive cough had been considered a tic by some and as psychogenic by others, Dr. Berman considered those diagnostic terms for the disorder. However, he commented that he observed no motor or vocal tics and no evidence of concerning emotional problems in those children. He reasoned that the ease of stopping the cough with a simple behavioral technique justified the term, habit cough. To this day, there is no reasoned justification for considering habit cough as a tic disorder, a tic cough, or psychogenic cough, terms that have been used by some without any basis.

A 1991 publication reported three boys and six girls, median age 11 years, who had been previously seen at the University of Iowa Pediatric Allergy and Pulmonary Clinic with histories of 2–5 months of a harsh barking nonproductive repetitive cough during all waking hours but absent when sleeping [6]. Coughing occurred in spasms and with unusual frequency. Medications prescribed by the referring physicians had been ineffective. The coughing was stopped within 15 min using an adaptation of Berman’s “art of suggestion”. Sustained benefit was documented by telephone contact a median of 3.6 years (ranging up to 9.4 years) after the clinic visit. Psychopathology was not apparent from a standardized psychological questionnaire. On the basis of that experience, suggestion therapy continued as the standard of care at the University of Iowa. 

### 3.2. Clinical Characteristics of Habit Cough

Thus, habit cough is not a new disorder despite the frequent lack of awareness regarding this disorder among much of the medical community. Just as described in the 1694 and subsequent publications, a respiratory illness is often an initiating factor at the onset of the repetitive cough [2]. Multiple case and series reports of children have since described a repetitive, daily nonproductive cough that is absent during sleep [5,6,7,8,9,10,11]. Habit cough is a distinctive syndrome recognizable and diagnosable by its unique clinical presentation. While the cough is most commonly described as barking or honking, home videos provided by parents demonstrate variability in the sound and frequency of the cough (https://youtu.be/U7p2B_Zt6AM, accessed 1 November 2022) Data from the University of Iowa clinic reported about 10% of the children having softer, throat-clearing type coughs [11].

While habit cough may not be recognized by a patient’s primary care doctor, parents generally readily recognize that their child’s cough is different from usual coughs with colds. A common comment from parents is that their child had a cold with a cough, and then after 1 or 2 weeks, the cough changed in sound and frequency. Routine spirometry and a chest X-ray provide sufficient reassurance that there is no physical basis for the cough. Further testing and therapeutic trials of medication result only in an iatrogenic component to the morbidity. The diagnosis is based on the uniqueness of the cough, its sound, frequency, nonproductive, and absence during sleep [11,12]. 

Occasionally, a child with successfully treated habit cough has a recurrence. While that data had not been systematically reported, our remote contact information included several parents reporting a recurrence of habit cough as much as a year later. Typically, it’s another viral respiratory infection that is followed by the recurrence of the habit cough. Children with recurrences self-managed their cough when reminded that they knew how to control the cough from their prior experience. One younger child, who had a return of habit cough a year later, required repeat suggestion therapy to stop the returned cough because she could not remember what she had previously done. 

### 3.3. Epidemiology of Habit Cough

In 2016, the diagnosis of habit cough was identified at the University of Iowa 140 times over a 20-year period from 1995 to 2014 based on information from the electronic medical record [11]. The average age was 10 years; 85% were between 8 and 13 years of age. The diagnosis was made by the frequent repetitive dry (nonproductive) cough that was absent once asleep. The absence once asleep despite a severe daily cough is a *sine qua non* for this disorder. A seasonal pattern with late spring and midsummer decreases in the frequency of diagnosis approximates the seasonal pattern of common viral respiratory infections.

**Figure 1:**
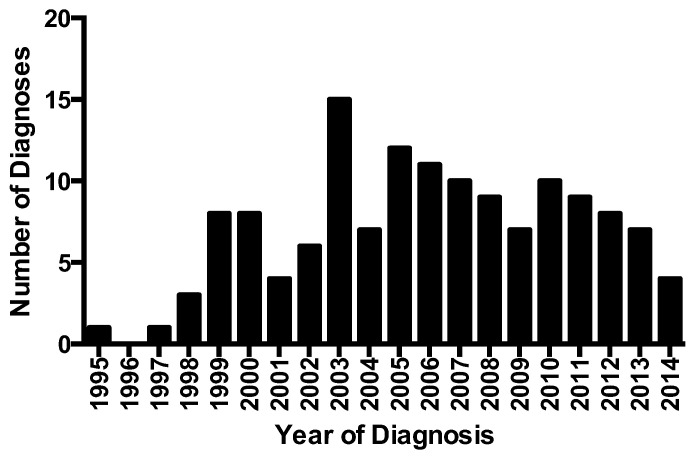
Annual diagnoses of habit cough by the faculty of the University of Iowa Allergy and Pulmonary Clinic from 1995 to 2014.

Using the same criteria for diagnosis of habit cough, a repetitive dry cough that was absent during sleep, Brompton Hospital in London, England reported the diagnosis of 55 children during a 6-year period, an average of nine per year [12]. Approximately the same frequency occurred at the Iowa clinic during the 12-year period from 2003 to 2014. The median age was 10 years at both institutions. For most of the children seen at these two institutions, cough had been present for periods from about a month to greater than 12 months prior to being seen. An increased frequency of habit cough diagnosis from 2.2 to 4.5 cases per year was attributed to a period of financial stress in Greece [13]. However, year-to-year variation of that magnitude also was seen during the 20 years at the University of Iowa (Figure 1).

More data on the prevalence of habit cough were a systemic evaluation of 346 children brought to centers in Australia, specifically for chronic cough. Protracted bacterial bronchitis, a cause of chronic cough primarily in infants, was present in 142 of the 346. Asthma was diagnosed in 55, and 48 had spontaneous resolution of the cough. Of the 101 other cases, 15 were diagnosed as “habitual” cough [14]. These data on the frequency of diagnosing habit cough among those presenting with chronic cough and the frequency with which habit cough is diagnosed at two major referral centers provide the best information we have regarding the prevalence of this functional disorder. A few physicians, predominantly pediatric pulmonologists, in 14 states in the U.S. and 10 other countries currently have indicated that they are diagnosing and treating habit cough in a manner similar to that described here.

The COVID-19 pandemic was associated with some community physicians indicating to us that they were seeing more children with habit cough. From the 11 children reported to us with characteristic habit cough post-COVID-19, it did not appear that habit cough post-COVID-19 differed from habit cough triggered by other viral respiratory infections. COVID-19 can cause an acute cough, but a chronic dry cough is reported to follow recovery that can last for months. This is described in an extensively referenced review [15]. The post-COVID-19 prolonged cough may or may not be accompanied by other long COVID-19 symptoms. Data in publications of respiratory post-COVID-19 follow-up in children are insufficient to ascertain the frequency with which some have the characteristics of habit cough [16,17].

### 3.4. Etiology of Habit Cough 

The cause of habit cough is an enigma. Why does it start? Why does it happen in some but not others? Why does it persist for months and even years? Cough hypersensitivity has been proposed as a hypothesis for the persistence of idiopathic or refractory chronic cough [18], However, evidence suggests that cough hypersensitivity may be both a cause and consequence of persistent coughing.

Mucosal biopsies obtained by bronchoscopy in adults with no medical explanation for their chronic cough showed inflammation [19]. Increased nerve density was reported in bronchial mucosal biopsies from adults with chronic unexplained cough [20]. The investigators of both of those studies suggested that the sustained daily coughing could be the cause of their findings. Thus, this neuropathic inflammation could be the cause of cough hypersensitivity and could be the stimulus for chronic daily cough. Essentially, this suggests a vicious cycle where repetitive daily coughing causes the mucosal pathology that is the nidus for the cough.

Clinical support for this explanation of persistent coughing is the common patient awareness of a “sensation” (sometimes called a tickle) in their airway that triggers their repetitive cough. Many patients have told us that the feeling that had been stimulating the repetitive cough only gradually fades over weeks even after cessation of cough. Thus, many patients continue to struggle with the stimulus to cough even after they have learned how to resist that urge. An analogy to which patients can relate is a mosquito bite that itches; the more you scratch it, the more it itches. It does not heal until the scratching stops. The desire to scratch is analogous to the desire to cough. In both cases, the cycle of itch-scratch and tickle-cough requires control of the sensation for healing to occur.

Clinical observations suggested that stress and anxiety contribute to habit cough in some patients. A description of stress associated with habit cough was described by Papadopoulou et al. [13]. However, association does not prove etiology. The stresses described by Papadopoulou et al. are not unique and occur also in children without chronic cough.

### 3.5. Treatment of Habit Cough

Habit cough has been effectively treated by various forms of suggestion therapy. This was first reported in 1966 by Berman [5]. It has been extensively used at the University of Iowa since 1975 [6,11]. A successful variation includes suggesting that a sheet tied around the chest enables cessations of cough [9]. Speech therapy [21] and cognitive behavioral therapy [13] have been proposed as treatments for habit cough, but no outcome data are reported for those. Hypnosis has been successfully used [10].

Suggestion therapy has now been used to the greatest extent for the successful treatment of habit cough. Increasing numbers of physicians and some nurse practitioners have found the approach to be effective. Although the provider of the suggestion therapy can alter the wording, major elements of suggestion therapy for habit cough include:

- Establish rapport with the patient and parents.
- Be honest and forthright with patients and parents.
- Tell the parents that you want to talk to the patient and ask them to sit quietly in the background, phones turned off.
- Body temperature water should be at the patient’s side or in hand to take small sips in anticipation of a cough.
- Explain the cough as a vicious cycle that started with an initial irritant that is now gone; it is now the cough itself that is causing the feeling that stimulates the coughing.
- Explain that cough is the body’s natural response to feeling that something is in their airway.
- Explain that the brain can control that response, but it takes a lot of concentration.
- Instruct the patient to focus on the provider of the suggestion therapy who is to keep up a relaxed soft patter.
- The patient is instructed to be aware when a cough may be coming and take a small sip of the water and hold the cough back.
- Begin with a request to hold the cough back for a defined period of time, beginning small and progressing as successes occur.
- Tell the patient that each second the cough is delayed makes it easier to suppress further coughing.
- Repeat expressions of confidence that the patient is developing the ability to resist the urge to cough: “It’s becoming easier to hold back the cough, isn’t it?” (Nodding your head generally makes the child nod their head in agreement.)
- When the patient shows that he or she is able to suppress the cough (usually after about 10 min), ask in a rhetorical manner, “You’re beginning to feel that you can resist the urge to cough, aren’t you?” (Say with an affirmative head nod.)
- Express confidence that if the urge to cough recurs, the patient can do the same thing at home (autosuggestion).

Autosuggestion should routinely be provided for patients to continue treatment at home. Autosuggestion refers to quiet sessions at home, with or without parental guidance depending on the patient’s preference, in which the patient performs their own suggestion by concentrating on holding back the cough using sips of body temperature water to “ease the irritation” causing cough. The use of online videos where suggestion therapy is successfully provided is helpful [22].

**Figure 2:**
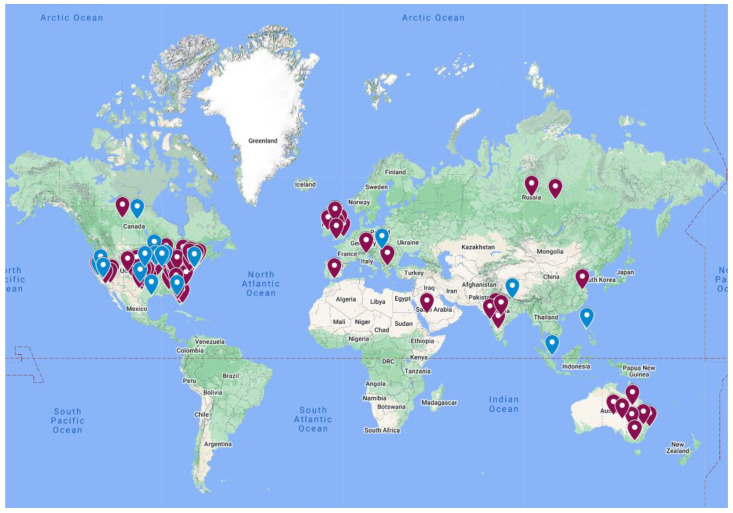
A map of 91 children (red dots) and 20 adults (blue dots) who informed us by email (via parents for children) of cough cessation from watching the video of a girl with chronic daily cough successfully stopped by suggestion therapy.

The first online video of habit cough treated by suggestion therapy was in February 2019 when one of the authors, MW, was contacted by the father of a 12-year-old girl (coauthor DB) with 3 months of a repetitive loud chronic barking cough that prevented her school attendance and disrupted the family milieu. Although the cough was absent during sleep, the cough repeated throughout all waking hours. A recording of that video was placed by her father on a website he created, www.habitcough.com (accessed on 1 November 2022), and on YouTube. That video has since become an important source of treatment (Figure 2).

### 3.6. Outcome of Habit Cough

A 15–30 min suggestion therapy strategy was used and taught during the 40 years of the corresponding author’s (MW) tenure at the University of Iowa Pediatric Allergy and Pulmonary Division [11]. The standard of care for habit cough at the University of Iowa Pediatric Allergy and Pulmonary Clinic was suggestion therapy provided by any of the faculty assigned to the clinic.

Suggestion therapy by direct contact and autosuggestion (self-suggestion) was more effective than just providing reassurance [23]. Of 85 children who received direct suggestion therapy in the clinic, 81 stopped coughing during 15–30 min of suggestion therapy. Three of the four who did not respond to suggestion therapy had apparent psychological problems that have been described previously (the fourth was not further evaluated) [24]. Autosuggestion instructions were provided to both those who did not receive suggestion therapy in the clinic and in addition to those who received direct suggestion therapy in the clinic since the continued desire to cough might last for days or weeks. 

Since the video became publicly available, 91 parents of children with habit cough and 20 adults from the U.S. and 15 other countries informed us by email that watching the video enabled cessation of coughing (Figure 2). Many found that repeated watching of the video was helpful. Eighteen of the ninety-one children additionally requested video contact with the doctor, author MW. A few requested the option of talking to the girl in the video, which was provided by video teleconference. The coughing stopped immediately in some, while sustained effort, rewatching the video, and repeated practice were described by others. The following emails illustrate these different responses:

This email was from a mother with whom we never had direct contact:

“*Our daughter, Riley, is seven years old (will be eight in May). A few months back, she had a really bad cold which led to a bad cough. After a few weeks the cold symptoms went away except for that cough. There was no stop to it. Just so much coughing*.

*I finally decided to just pull your video up on YouTube and we all sat there and watched it. It was very emotional for all of us and at the end Riley was in tears (we all were). We hugged for a time. She said to us “I can hold the cough back. And the…THE COUGHING STOPPED! Like turning off a switch. For four days now, I have not heard her cough except for a few random ones here and there. The cough is GONE!*”

The following email is from the mother of a 14-year-old boy with a history of coughing about two times per minute during waking hours. This continued for more than a month after the clinical symptoms of a viral respiratory infection. Suggestion therapy was provided by MW via remote video conferencing:

“*He is doing so much better. He went back to school this week. The first 2 days he had to really work to control the cough and had a few bouts of having a difficult time of doing so. However, the last few days he hasn’t coughed at all and the urge is less and less*.”

These two cases demonstrate the variability in response to suggestion therapy, immediate cessation of coughing or motivated daily practice to accomplish cessation of cough.

We have no way of estimating how many others have watched the video and never contacted us. Physicians in the U.S., Ireland, Israel, and Australia have reported to us that they have referred habit cough patients to that video and received feedback from parents of chronic cough cessation, but we have only the attestation of the physician for those communications.

### 3.7. Clinical Course of Untreated Habit Cough

In 1991, Mayo Clinic in Rochester, Minnesota reported an 18-year follow-up of 60 children in their medical record archives with “childhood involuntary cough syndrome” consistent with habit cough [25]. The ages of the children were similar to those reported at the University of Iowa [11]. Since no treatment beyond diagnosis and counseling was given, this essentially provided the natural history for untreated habit cough. Cough spontaneously resolved only after a mean duration of 6.1 months after diagnosis in 44 (73%) of the 60. Sixteen (27%) were still coughing a mean of 5.9 years from the time of diagnosis.

At the Brompton Hospital in London, parents were provided only “simple reassurance [12]”. A follow-up for 1.9 years was reported in 39 of the 55 children who they were able to contact. Spontaneous resolution of cough was reported in 59% of the 39 within 4 weeks; the others had persistent coughing for longer periods, especially if there was skepticism by the parents regarding the diagnosis.

Further evidence of the potential duration of habit cough was obtained from four adults among the remote contact patients. Their ages ranged from 23 to 32. They described 8–15 years of chronic cough that had begun when they were adolescents. Their clinical characteristics were consistent with habit cough, i.e., daily repetitive nonproductive cough that was absent when asleep. The available data, therefore, show eventual spontaneous resolution for chronic cough in many, but some continue with a persistent cough for years. Consequently, when encountering a child with habit cough, a wait-and-see approach for spontaneous resolution is not a justifiable strategy.

### 3.8. When Suggestion Therapy Is Not Effective

One of the authors of the article, RA, is a pediatric pulmonologist with skills in medical hypnosis that have been used to successfully treat habit cough [10]. Results of hypnosis for habit cough have found that approximately half of all patients with habit cough who use hypnosis initially resolve their cough immediately after the first application of hypnotic relaxation. Most of the others with typical habit cough who are helped with hypnosis resolve their cough within a month.

Of ten patients from the remote contact source who failed to respond to suggestion therapy, only two improved with hypnotic relaxation alone. Four of those ten patients improved after the use of hypnosis to promote interactions with the part of the mind of which one is not fully aware. Although subconscious, this can nonetheless influence actions and feelings. The use of this strategy is demonstrated in a case study described in *Changing Children’s Lives with Hypnosis* [26].

An example of a patient benefiting from sessions with a medical hypnotist was Stella. Stella was a delightful 12-year-old girl from Texas whose father contacted the author MW in February 2022. Her ambition was to be an actress. She coughed about every 3–5 s continually throughout all waking hours but did not cough when sleeping. Despite several remote conversations where she was fully cooperative and highly motivated to stop, there was no progress. Local hypnosis and counseling were arranged by her father at the recommendation of author RA. Six months later her father described the successful cessation of Stella’s habit cough after eight sessions with a local hypnotist selected on the basis of being skilled in medical hypnosis for children.

Interestingly, the few children resistant to suggestion therapy appeared to differ from those who responded to suggestion therapy. The “tickle,” the feeling in the throat that was typically present in patients with habit cough, was not present in these children. They did not notice any precough tickle or stimulus. They also had persistent very high cough frequency, cough every few seconds, without the greater variation from distraction commonly present in others. In some, the cough seemed to occur with every exhalation.

What are we to think about these markedly different responses to suggestion therapy and hypnosis in this small minority of children diagnosed with habit cough? Are they totally different disorders or, as with many diseases, are there different phenotypes of habit cough?

## 4. Discussion

Chronic cough is a horribly unpleasant ailment. Coughing hundreds of times every hour of their waking lives has a profound impact on their quality of life. Muscular pain, headache, and depression are common in children and adults with this functional cough. Habit cough has been in the medical literature for over 300 years. The children from the University of Iowa and the remote contact data set we have accumulated provide evidence for a serious disorder that, while not common, is also not rare. Two major referral centers, one in the U.S. and one in England, reported an average of nine children with habit cough per year in their clinics.

### 4.1. What Is the Current Status of Identifying and Treating Habit Cough

Just as Dr. Berman recognized and treated habit cough in his 1966 publication [5], other physicians have recognized incessant coughing without an apparent cause as a habit disorder and used their clinical skills to provide their own approach to break the habit [9,10]. Publications over the past few years [11,22,24] and invited presentations to 12 pediatric pulmonary divisions in the U.S. and pediatric pulmonary conferences in London and Taipei have resulted in an increasing number of physicians who provide recognition and competent treatment for habit cough. A link for the recording of one of those presentations at the National Children’s Hospital is available at www.milesweinberger.com (accessed on 1 November 2022).

Despite the increased awareness of some physicians, primarily pediatric pulmonologists, frustrated parents continue to contact us because their medical providers do not recognize or know how to treat the “horror” cough from which the child is suffering. The awareness of the medical community about habit cough needs to be substantially increased.

### 4.2. Barriers to Diagnosis and Treatment

Why the difficulty in recognizing and treating a disorder that a Boston allergist reported as responsive to “the art of suggestion” in 1966? [5]. False beliefs about habit cough have been a major barrier (Table 1).

A concerning barrier to recognition and treatment of habit cough is that the perceptions of some physicians are not consistent with the morbidity caused by this disorder. Perceptions that habit cough was not as important as physical disorders were occasionally expressed as were concerns about the time required for suggestion therapy. However, despite the absence of a physical cause, these children have serious morbidity that is readily treatable. As to having the time, that is a lame comment. When necessary, a physician makes the time to provide appropriate care for a patient in need.

A debate on the subject of terminology for this cough disorder was arranged by Richard Irwin, a cough specialist and editor of the journal *Chest*, between one of the authors of this article (MW) and a neurologist, Tamara Pringsheim, MD [36]. Skepticism regarding habit cough also was expressed by Dr. Peter Dicpinigaitis, current editor of the journal *Lung*. When provided a description of habit cough from one of the authors, he stated in an email, “I’m not a fan of science fiction”.

While cough may occur in patients with Tourette’s syndrome, our experience with five patients who had Tourette’s syndrome found that cough was in addition to Tourette’s typical symptoms of motor tics and vocalizations. One patient was Jacob, an 11-year-old boy whose father and he both had typical symptoms of Tourette’s. Following symptoms of a common cold, he developed a severe repetitive cough, absent during sleep, for a month prior to contact with us. His cough responded to suggestion therapy without any effect on his tics or occasional vocalizations. Father, who had lived with his own Tourette’s symptoms indicated that the 1 month of cough experienced by his son was not related to symptoms of Tourette’s. Another example of the lack of association of habit cough and Tourette’s was Soraya, a 10-year-old girl with typical symptoms of Tourette’s since age 3. After symptoms of a cold, she had repetitive coughing, multiple times per minute, absent only during sleep, for 2 months. She responded readily to suggestion therapy by proxy. A recurrence a year later after another cold was stopped within a week after suggestion therapy was provided by remote video conferencing.

An unfortunate consequence of this diagnostic dialectic is the experience of Tom, a 16-year-old boy with 3 years of chronic cough. In 2020, a neurologist at Children’s Hospital of Philadelphia attributed his cough to Tourette’s syndrome, a diagnosis that stayed with him until suggestion therapy provided by remote video conferencing enabled him to control his cough more than 2 years later. Three days after the remote video conferencing, Tom reported coughing had gone from thousands per day to no more than ten. Ten days later, Tom was continuing to be free of his former daily repetitive cough with no symptoms of Tourette’s.

Thus, our experience has been that motor tics were not associated with habit cough. Cough was distinct from the echophenomena of Tourette’s. While there have been published case reports of chronic cough attributed to Tourette’s [37,38], our experience indicates that when typical symptoms of Tourette’s are present, a chronic repetitive dry cough present during most waking hours that is absent during sleep is likely a separate clinical problem and not a component of the Tourette’s.

Because of the nature of habit cough and its cure by a simple behavioral technique, suggestion therapy, a degree of incredulousness has inhibited more widespread attention to this disorder. Newspaper articles hoping to educate the public have been few. The Daily Beast on 29 April 2019 was the only story about the habit cough and its cure by suggestion in the U.S. An article in the *Scotsman*, a major Scottish newspaper, on 21 March 2021 described the cure of habit cough in an 11-year-old girl who had been coughing for a year. She stopped coughing by watching the video of suggestion therapy provided to a 12-year-old girl. Further publication in the health section of major newspapers would distribute information that enables parents to seek earlier diagnosis and treatment. 

### 4.3. Guidelines for Chronic Cough from Professional Societies

A guideline for the diagnosis and treatment of chronic cough in adults and children was published by the European Respiratory Society in 2020 with 18 authors [39]. Recommendations were for therapeutic trials of inhaled corticosteroids with and without a long-acting bronchodilator, an antileukotriene, and a macrolide. Low-dose morphine and gabapentin were suggested for adults. An antibiotic trial was recommended for children. The recommendation was indicated as conditional with the level of evidence rated low to moderate. This guideline addressed gastrointestinal reflux and postnasal drip, also known as upper airway cough syndrome, two disorders some cough specialists have regarded as contentious. Habit or tic cough was suggested to be labeled somatic cough disorder, and the guideline indicated that the diagnosis should only be made after an extensive evaluation that rules out tic disorders and uncommon causes of chronic cough. That guideline did not recognize that four major referral centers, the University of Iowa, SUNY Upstate Medical University, Rainbow Children’s Hospital in Cleveland, and Brompton Hospital in London had been diagnosing habit cough for years based on the unique clinical presentation without extensive evaluation [11,12].

A guideline published in 2020 by an Expert Panel in the journal *Chest* focused on chronic cough in children [27]. The report acknowledged that many questions addressed in systematic reviews of chronic cough did not contain high-quality studies or evidence. Gastroesophageal reflux and postnasal drip were not supported as being of concern in children. Emphasis was placed on the importance of identifying various diseases that cause cough, such as tuberculosis, pertussis, and protracted bacterial bronchitis. While habit cough, also known as tic cough or somatic cough syndrome, was discussed, a major characteristic of habit cough was discounted. Specifically, the expert panel concluded that “the presence or absence of cough when sleeping should not be used to diagnose or exclude psychogenic or habit cough”. This was inconsistent with virtually every publication describing absence when asleep as a characteristic of habit cough [4,5,6,7,8,9,10,11].

### 4.4. Strengths and Limitations of This Report

The strength of this report is the presentation of the extensive past and present experiences with habit cough of the authors, the provision of data related to the clinical course of children not treated with suggestion therapy, and the observations that suggestion therapy stops chronic cough in a substantial number of children and some adults with this disorder. Suggestion therapy is effectively provided by direct contact, indirect contact by remote video conferencing, and by proxy using a video of an effective session of suggestive therapy.

A limitation of the report is that all of our data are observational with no comparative control group. Although we serendipitously found habit cough responsive to suggestion therapy in 20 adults, we do not know what portion of the 40% of adults with idiopathic or refractory chronic cough have habit cough [29,30]. 

## 5. Conclusions and Recommendations

Habit cough is a distinctive disorder characterized by a repetitive daily nonproductive cough that generally persists during all waking hours but is absent once asleep. This description is the basis for diagnosis. Major medical centers that have routinely made the diagnosis of habit cough primarily on that clinical presentation include 140 children over 20 years at the University of Iowa, 56 children over 5 years at SUNY Upstate Medical University and Rainbow Children’s Hospital in Cleveland, and 55 children over 6 years at Brompton Hospital in London, England. Treatment by suggestion therapy is extraordinarily successful and should now be the standard of care for children with this disorder.

The provision of suggestion therapy can potentially be provided by the patient’s physician that makes the diagnosis. The characteristic clinical presentation of a repetitive dry cough that is absent when asleep is generally sufficient for diagnosis. While a chest X-ray and spirometry may help provide assurance for the family of the absence of an organic cause, there is rarely an indication for more testing or therapeutic trials. Suggestion therapy should be approached with positivity. Suggestion therapy is not something to be tried, it is to be executed with confidence.

There is evidence that habit cough also can be a cause of chronic cough in adults. Medical books more than 300 years ago [2,3] described a habitual severe cough in adults, and one describes the absence once asleep, a *sine qua non* for the diagnosis of habit cough [2]. Emails to us from 20 adults describe the same clinical pattern of cough and the same response to suggestion therapy as in children with this disorder. The prevalence of habit cough in adult populations with idiopathic or refractory chronic cough warrants investigation. Those with a clinical pattern consistent with habit cough should be considered for a controlled clinical trial of suggestion therapy.

## Figures and Tables

**Table 1:** Popular factoids and actual facts regarding habit cough.

**Children with habit cough exhibit *la belle indifference*.**
It has been suggested that children with habit cough appear to not care about their cough. While children may adapt to living with a chronic cough, few if any have appeared to us content about their cough. It is life-altering for the child and their family and, if asked, the children express concern about their cough. They are not indifferent to their cough.
**School phobia can precipitate or perpetuate habit cough.**
This claim was made in a 2020 Chest publication [27]. The reference used to support that claim was a case series of adults at a psychiatric facility [28]. While there are occasional patients with school phobia as a factor, our experience does not support the assertion that school phobia is a common concern as a cause of habit cough. We have observed that the children with habit cough are generally concerned about missing school. Those who had been out of school because of the cough are generally enthusiastic about returning.
**Habit cough is exclusively a pediatric disorder.**
We have been contacted by 20 adults from eight countries with chronic cough who reported to us by email that their coughing was stopped by suggestion therapy from viewing the video made available by coauthor DB (see Figure 2). In addition, three adults had their chronic cough resolved by hypnosis therapy performed by coauthor RA. It is not known how many with habit cough are among the 40% of adults with idiopathic or refractory cough [29,30]. Our serendipitous experience identified 20 adults with the same clinical characteristics as the children with habit cough who described cessation of cough by suggestion therapy. Interestingly, Peter Dicpinigaitis, editor of the journal *Lung*, states in a publication that among 1000 patients he has never seen psychogenic cough (a euphemism for habit cough) [31]. However, Dr. Kefang Lai described 23 adults that he termed “somatic cough syndrome” (another euphemism for habit cough) [32]. Other case reports of psychogenic cough in adults also has been described [33,34].
**Habit cough diagnosis requires that all other causes of cough are eliminated.**
While this has been stated without support in guidelines for chronic cough, habit cough has a sufficiently distinctive clinical presentation that major centers experienced with this disorder make the diagnosis with little testing or therapeutic trials. Efforts to examine for other causes of cough only add to the morbidity and cost [11,12]. Treating habit cough early in the course of its clinical presentation can prevent unnecessary and expensive investigations that delay resolution.
**Habit cough is a tic disorder, a *forme fruste* of Tourette’s syndrome.**
This was claimed by Dr. Alyn Morice, a prominent cough specialist, who wrote in an email, “You are describing, but failing to recognize, *a forme fruste* of Tourette’s syndrome”. However, cough is not supported as a usual finding in Tourette’s 1884 publication [35]. Moreover, during interviews with parents of children with Tourette’s, cough was readily recognized as cough and not a tic and was regarded as separate from the symptoms of Tourette’s in their child.

## Data Availability

Data in the form of parent of patient email communications are available for purposes of further research of habit cough.
